# Non-Rigid Registration of Liver CT Images for CT-Guided Ablation of Liver Tumors

**DOI:** 10.1371/journal.pone.0161600

**Published:** 2016-09-09

**Authors:** Ha Manh Luu, Camiel Klink, Wiro Niessen, Adriaan Moelker, Theo van Walsum

**Affiliations:** 1 Biomedical Imaging Group Rotterdam, Departments of Radiology & Medical Informatics, Erasmus MC, Dr. Molewaterplein 50/60, Rotterdam, The Netherlands; 2 Department of Radiology, Erasmus MC, Dr. Molewaterplein 50/60, Rotterdam, The Netherlands; Academia Sinica, TAIWAN

## Abstract

CT-guided percutaneous ablation for liver cancer treatment is a relevant technique for patients not eligible for surgery and with tumors that are inconspicuous on US imaging. The lack of real-time imaging and the use of a limited amount of CT contrast agent make targeting the tumor with the needle challenging. In this study, we evaluate a registration framework that allows the integration of diagnostic pre-operative contrast enhanced CT images and intra-operative non-contrast enhanced CT images to improve image guidance in the intervention. The liver and tumor are segmented in the pre-operative contrast enhanced CT images. Next, the contrast enhanced image is registered to the intra-operative CT images in a two-stage approach. First, the contrast-enhanced diagnostic image is non-rigidly registered to a non-contrast enhanced image that is conventionally acquired at the start of the intervention. In case the initial registration is not sufficiently accurate, a refinement step is applied using non-rigid registration method with a local rigidity term. In the second stage, the intra-operative CT-images that are used to check the needle position, which often consist of only a few slices, are registered rigidly to the intra-operative image that was acquired at the start of the intervention. Subsequently, the diagnostic image is registered to the current intra-operative image, using both transformations, this allows the visualization of the tumor region extracted from pre-operative data in the intra-operative CT images containing needle. The method is evaluated on imaging data of 19 patients at the Erasmus MC. Quantitative evaluation is performed using the Dice metric, mean surface distance of the liver border and corresponding landmarks in the diagnostic and the intra-operative images. The registration of the diagnostic CT image to the initial intra-operative CT image did not require a refinement step in 13 cases. For those cases, the resulting registration had a Dice coefficient for the livers of 91.4%, a mean surface distance of 4.4 mm and a mean distance between corresponding landmarks of 4.7 mm. For the three cases with a refinement step, the registration result significantly improved (p<0.05) compared to the result of the initial non rigid registration method (DICE of 90.3% vs 71.3% and mean surface distance of 5.1 mm vs 11.3 mm and mean distance between corresponding landmark of 6.4 mm vs 10.2 mm). The registration of the preoperative data with the needle image in 16 cases yielded a DICE of 90.1% and a mean surface distance of 5.2 mm. The remaining three cases with DICE smaller than 80% were classified as unsuccessful registration. The results show that this is promising tool for liver image registration in interventional radiology.

## Introduction

Primary liver cancer is one of the most fatal cancers. The 5-year survival rate of patients without treatment is 15% [1–3]. So far, the preferred treatment is liver surgery. However, not all the patients are eligible for such an invasive procedure. Minimally invasive approaches such as radiofrequency ablation (RFA), microwave ablation, radiotherapy, chemoembolization, and high-intensity focused ultrasound are alternatives in case surgery is not an option [1, 4]. In chemoembolization drugs are brought to the tumor via a catheter in the arterial system, and in case of ablation, a needle is introduced percutaneously into the tumor. Such treatments, being minimally invasive, require image-guidance during the intervention. These techniques are suitable for patients with tumors detected in early stages (< 3 cm in diameter) [4, 5].

**Fig 1:**
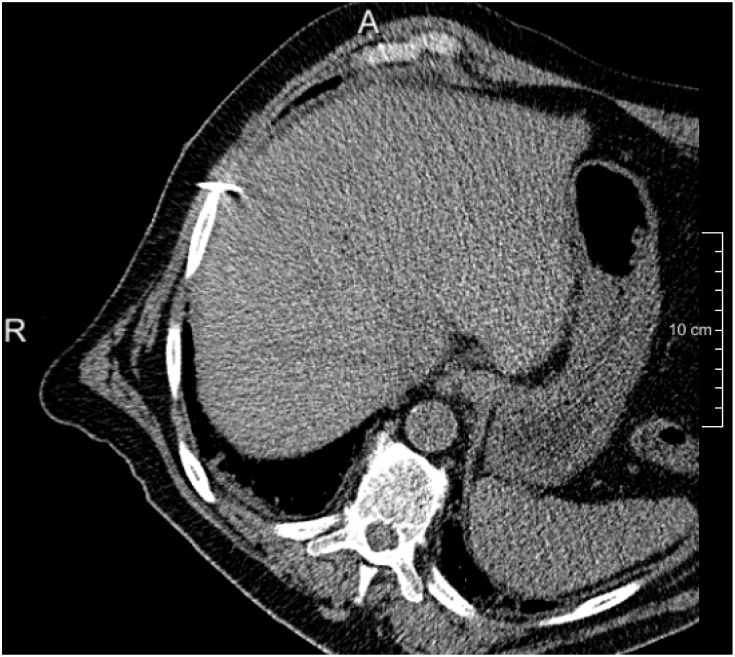
An example of intra-operative image, note the rotation of the patient required for safe needle insertion from the side.

Contrast enhanced MRI or contrast enhanced CT is performed in the diagnostic phase, to assess the liver cancer stage [4, 6] and to extract information such as tumor location and size. CT and US are commonly used during the ablations to guide the needle to the target tumor. Unfortunately, the tumor is not always visible using these imaging modalities. As a consequence, the interventional radiologist mentally maps the location of the tumor from the diagnostic (contrast-enhanced) images to the intra-operative images. This procedure is inconvenient, time-consuming and potentially inaccurate, as the human ability to mentally map a 3D object into a 3D space is limited [7, 8]. Furthermore, after initial needle insertions, CT scans with a limited number of slices are acquired, to check the needle position that is advanced to the tumor step-wisely. The limited field-of-view (FOV) hampers accurate mentally mapping of the diagnostic information. Therefore, the purpose of our work is to improve image guidance by projection of the liver tumor during the intervention. This is achieved by semi-automatically spatially aligning the pre-operative contrast-enhanced CT image with the intra-operative CT images, such that the tumor can be visualized in the intra-operative CT images (see Fig 1).

The challenges of spatially aligning (registering) diagnostic and intra-operative CT images are:

1. Deformations of the liver because of difference in patient pose
2. Change in position of the liver w.r.t. other organs because of respiration
3. Change of tissue surrounding the liver, e.g. changes in gallbladder filling or air in the intestines
4. Limited number of slices in the scans that are taken during needle insertion

Additionally, for our application, the registration should be sufficiently fast to be used in an interventional setting.

Some studies already reported on approaches to improve image guidance during ablations. Rieder et al. (2014) [9] developed a tool to segment the tumor in the intra-operative images. This method requires a contrast-enhanced acquisition to highlight the tumor and vessel. No quantitative evaluation results have been reported in their study and the use of contrast agent for imaging at the start of the intervention is generally not preferred, instead the contrast-enhanced acquisition is used to check the ablation or even cancelled in case of renal insufficiency. Archip et al. (2007) [10] introduced a registration framework using a finite element based method (FEM) to fuse diagnostic MRI to intra-operative CT of the liver for RFA. Elhawary et al. (2010) [6] used a non-rigid registration method with a B-spline based non-rigid transformation model to align diagnostic MRI image to intra-operative CT in cryoablation of liver tumors. To the best of our knowledge, there has been no study addressing the problem of registering diagnostic and intra-operative image data for the purpose of guiding needle insertion in ablation procudures using CT imaging only.

Our contribution thus is the development and evaluation of a method for improving image guidance in CT-guided ablation procedures by aligning pre-interventional diagnostic images with intra-operative images. This approach enables the integration of the tumor annotation from the diagnostic images in the intervention. To this end, we propose a two-stage non-rigid registration approach. The first stage is an initial registration, the purpose of which is to compute large deformations, including an optional user-guided refinement to locally improve the alignment. This registration approach allows for overlaying the tumor information in the initial CT scan made at the start of the intervention. In the second stage, the limited field-of-view intra-operative image with the needle in place is registered to the initial intra-operative CT image. Combination of the results of both stages enables tumor site integration in the limited field-of-view intra-operative images. The method is quantitatively evaluated on 19 datasets.

Organization of this paper is as follows: in the next section, we introduce our method. Section III presents our experimental setup and the evaluation framework, as well as the results of our experiments. In Section IV, these results are discussed, and conclusions are drawn in Section V.

## Method

The data was anonymized and de-identified prior to analysis by the Trial Office. The Erasmus MC METC (Institutional Review Board) declared that this study, because of its retrospective nature, does not need IRB approval.

### Image registration

Image registration is a powerful technique in medical image processing, and there are many publications on registration approaches and their application to specific medical imaging problems [11–15]. In image registration, a spatial transformation between two images, the fixed (target) image and a moving (source) image is determined [16, 17]. Mathematically, it is commonly written as an optimization process which finds the transformation **T(x)** = **x** + **u(x)** that relates the two images such that the transformed moving image *I*_*M*_(**T(x)**) spatially matches the fixed image *I*_*F*_(**x**) at every position of **x**, where a metric *M*(*I*_*F*_(**x**), *I*_*M*_(**T(x))**) is used to quantify the quality of the match. Several transformation models can be used for the transformation **T(x)**, ranging from simple translation via rigid and affine to high-dimensional non-rigid transformation models. For 3D non rigid registration, a common representation of the deformation field **u(x)** is a cubic B-spline [12, 16] which is parameterized by parameter vector *μ*.

The metric *M* is often a similarity metric; Mutual information (MI) and normalized cross correlation (NCC) are typical metrics used in a cost function. In most cases, metrics are negated to obtain a dissimilarity metric, and the registration is turned into a minimization problem. Registration thus can be viewed as finding the set of the parameter $\hat{\mu }=argminC\left(\mu ;I_{F},I_{M}\right)$ where *C*(*μ*; *I*_*F*_, *I*_*M*_) is the cost function related to the similarity metrics [16].

### Two-stage registration approach

**Fig 2:**
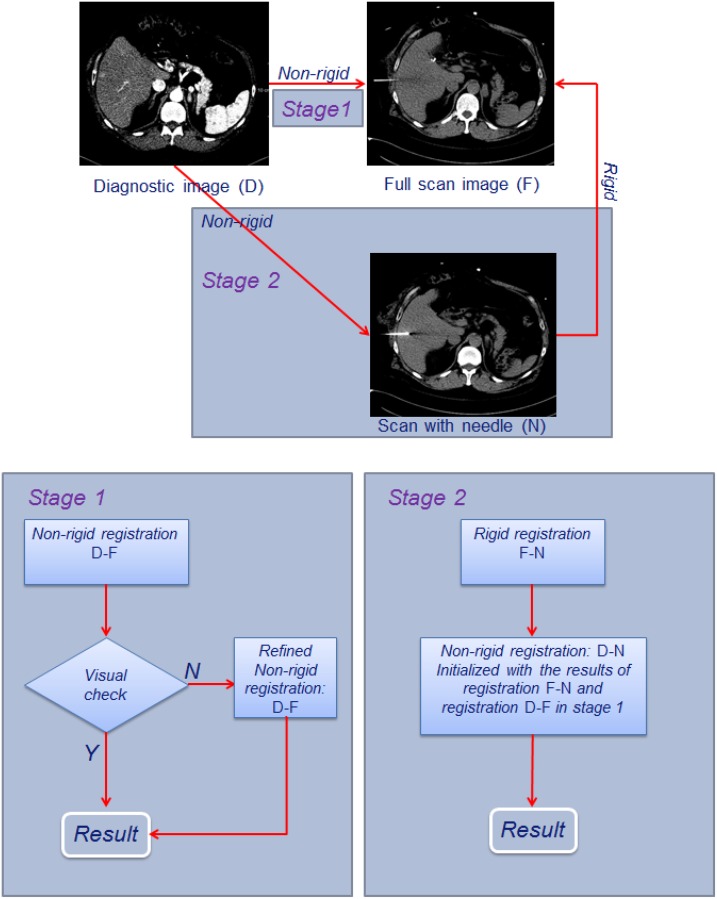
Registration scheme of the diagnostic contrast enhanced CT image D and the intra-operative CT images: Stage 1 is registration between the image D and the full operative image F; and stage 2 is the registration between the image D and the operative image N.

In clinical practice of CT-guided percutaneous ablation, the interventional radiologist acquires a CT scan of the complete liver at the start of the intervention to determine the target location, and to determine the entrance point of the needle on the patient’s skin. Subsequently, during needle introduction, CT-images with a limited number of slices are acquired to assess the position and orientation of the needle. Based on this workflow, we propose to register the diagnostic image to the CT image with needle in situ in two stages: first the diagnostic image (D) is registered to the first complete liver operative image (F), which enables integration of the tumor in the planning CT image, and subsequently the initial operative image is registered to the limited field-of-view image with the needle (N), (see Fig 2). The main reason for this two-step approach is that achieving a direct registration of the image with needle to the diagnostic image is infeasible: differences in patient positioning and breathing state require a non-rigid registration, and the limited field of view hampers an accurate direct registration directly to the diagnostic image. However, during the intervention, the patient is sedated and kept in a stable position, and breathing is shallow. So a rigid registration is sufficient to compensate the drift motion of the liver caused by shallow breathing of patient during intervention. Pilot experiments showed that the CT image with the needle can be registered to the first operative CT image relatively easily. Non-rigid registration of image D to image N (NR: D-N) is performed by initializing with the combined result of a rigid registration of image F and image N (R: F-N) and a non-rigid registration of image D to image F (NR: D-F).

In our study, we used MI as the metric to measure the image alignment, and also include a regularizer for the deformation field:

$$C\left(\mu ;I_{F},I_{M}\right)=-MI\left(\mu ;I_{F},I_{M}\right)+\alpha R\left(\mu \right)\;,$$ (1)

where *R*(*μ*) is a regularization term which constrains the non-rigid deformation and *α* is a weight term which balances the similarity metric *MI*(*μ*; *I*_*F*_, *I*_*M*_) and the regularization term *R*(*μ*) [11].

In practice, because the histogram is binned, the mutual information is defined as:

$$MI\left(\mu ;I_{F},I_{M}\right)=\sum_{m\in {Ł}_{M}}\sum_{f\in {Ł}_{F}}p\left(\mu ;f,m\right)\frac{p(\mu ;f,m)}{p_{F}\left(f\right)p_{M}\left(\mu ;m\right)}\;,$$ (2)

where *L*_*F*_ and *L*_*M*_ are sets of regularly spaced intensity bins of histograms of the fixed image and the moving image correspondingly; *p* is the discrete joint probability of the image intensities of the two images; *p*_*F*_ and *p*_*M*_ are the marginal discrete probabilities of the fixed image and the moving image intensities correspondingly.

### Stage one: initial registration

**Fig 3:**
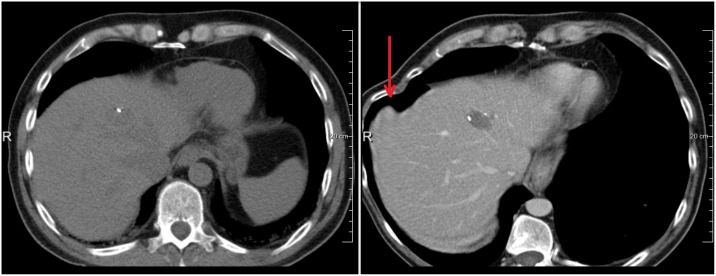
Example of incorrect deformation at the edges of the liver in case the liver boundary in the intra-operative image is unclear. The arrow points to the region with incorrect deformation.

The major challenge in this procedure is to accurately register the diagnostic image D with the initial intra-operative image F. Therefore, we propose a registration approach that permits user interaction to correct the registration. Initially, a non-rigid registration is applied to obtain alignment. The registration method uses a liver mask which covers the liver and excludes unrelated neighbouring organs such as the heart, the lungs, etc. from the registration. Incorrect registrations, with unrealistic deformation, may occur in this registration because of the large pose changes and changes in breathing state between the diagnostic and intra-operative images, especially near the liver boundary (see Fig 3).

For the registration, we used the framework of a previous study, where pre- and post-interventional images were registered for therapy assessment [18]. The framework performs registration in a multi-resolution strategy; however, in contrast to our previous work, we now choose MI as similarity metric because the intra-operative images are often non-contrast enhanced.

### Stage one: user-guided refinement

An optional user-guided registration step may be applied next to further improve the registration. The user may annotate the region of incorrect registration by clicking one or more points in the region in the diagnostic image where the incorrect deformation occurs. In practice, these regions have a large non-rigid deformation component, and the refinement method is focused on reducing this erroneous non-rigid deformation. The annotated points serve as seed points for a subsequent dilation (1x1x1 cm kernel). The combined dilated regions form the area *C*, which is used as a coefficient mask in the registration, i.e. non-rigid components in the transformation inside the area *C* are penalized by using the local rigidity term introduced by Staring et al. (2007) [19], and also recently adapted in previous work [18] to improve the smoothness of the transition areas between local rigidity areas and non-rigid transformation areas. Specifically, the regularization term *R*(**T**) in Eq 1 is replaced by the following term, which is defined locally by coefficient mask *C*:

$$\begin{array}{c} P^{rigid}\left(T;I_{M}\right)=\frac{1}{\sum_{x}c\left(x+T\left(x\right)\right)}\times \\ \sum_{x}c\left(x+T\left(x\right)\right)\times \left\{c_{AC}\sum_{k,i,j}AC_{kij}{\left(x\right)}^{2}+c_{OC}\sum_{i,j}OC_{kij}{\left(x\right)}^{2}+c_{PC}PC{\left(x\right)}^{2}\right\}\;, \end{array}$$ (3)

where the weights *c*_*AC*_, *c*_*AC*_ and *c*_*PC*_ balance the three terms: affine term *AC*_*kij*_(**x**), orthonormality term *OC*_*kij*_(**x**) and properness term *PC*(**x**); and *c*(**x**) ∈ [0, 1] is a user-predefined coefficient of mask *C* at location **x** (Staring, 2007).

### Stage two: non-rigid intra-operative image registration

In stage 2, the intra-operative image with the needle N is rigidly registered to the full intra-operative image F. Both the diagnostic image and the limited field-of-view image are registered to the full intra-operative image. In order to avoid an accumulation of registration errors, a non- rigid registration is subsequently applied to register the diagnostic image to the intra-operative image with the needle. Note that, after initial alignment using transformation from the registrations, the diagnostic image and the intra-operative image with the needle are already relatively well spatially aligned, and thus a subsequent non-rigid registration is feasible. In case that the initial registration of image D and F needs a refinement registration, the annotated mask is also applied to the registration in stage 2. The tumor annotated by using the diagnostic images is transformed to the intra-operative image with the needle by using transformation of the new registration, enabling an integrated visualization of the needle and the tumor area.

## Experiments and Results

### Image data

Abdominal diagnostic and intra-operative anonymized CT scans of 24 patients were randomly selected and from patients that underwent abdominal CT scanning and CT-guided RF ablation in the Erasmus MC in 2014 and 2015. All of the data were acquired by a Siemen CT scanner, 64-rowmultidetector, pitch 0.8, rotation time 0.5s. From the diagnostic scans, the contrast enhanced images were selected for the study. The images have a resolution of 0.56 mm x 0.56 mm to 0.89 mm x 0.89 mm, 1-10 mm slice spacing, 1-2 mm slice thickness, 512x512 pixel in plain resolution. Image acquisitions were performed according to the standard clinical protocol: 60 seconds after the injection of 120 cc intravenous contrast agent. The intra-operative images were acquired with a tube voltage of 100-120 KV and a tube current of 172—288 mAs with resolution of 0.56 mm x 0.56 mm to 0.90 mm x 0.90 mm and slice spacing of 2.5-5 mm. Of these images, 13 are non-contrast enhanced images; 11 are with contrast enhanced with 80-120 cc intravenous contrast agent. The main reason for the contrast enhanced images is the inability to mentally match tumor location on non-contrast CT with previous contrast enhanced diagnostic CT. Furthermore, in 12 out of the 24 cases, the patient was rotated (30-50 degrees) w.r.t. a supine position on the CT table, to provide access to the appropriate needle introduction site, see e.g. Fig 1. In general, the field of view of the 3D abdominal images is larger than the region of interest i.e. the liver. Hence, to reduce processing time for image registration, in a pre-processing step, we manually cropped the images to the liver. We randomly picked five cases for pilot experiments, leaving 19 cases for the final analysis.

### Manual segmentation of the tumor and liver mask

**Fig 4:**
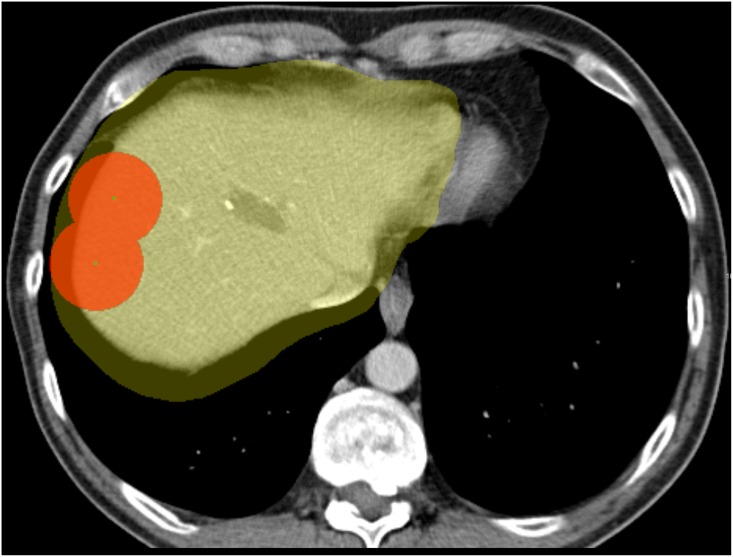
The coefficient mask (in red) is used to penalize the rigid deformation of the liver inside the liver mask (in yellow).

The liver tumor was manually segmented in the diagnostic image. The liver masks of the diagnostic image and the intra-operative image, which are used in the registration to limit the computation of the similarity metric to the liver region only, were annotated by drawing approximately 10 smooth contours slightly larger than the liver (see Fig 4). Note that the liver masks do not need to be accurate liver segmentations; automated liver segmentation methods [20] could be used as a substitute. Additionally, the liver was accurately segmented manually for evaluation purposes (see Section evaluation metric); this segmentation was not used in the method.

### Registration software and parameter settings

In this study, we used Elastix, a free registration software package developed by Klein and Staring [19] which is available at elastix.isi.uu.nl. The parameter file of the registration can be found at the elastix database of parameter setting which contains the relevant parameter settings. The important settings are as following: If the patient on the CT table was rotated during the intervention, the images were manually rotated (along the z-axis) such that the spines on both images have approximately the same orientation. A multi-resolution approach was utilized to handle the variation in the size of the patient’s liver as well as image resolution. The initial alignment of registration is based on center of mass of the liver mask. The four resolutions of the B-spline grid are set to [80 40 20 10] mm. The weight parameters for rigidity are set as (RigidityPenaltyWeight 0.1 0.1 0.1 4.0), (LinearityConditionWeight 100.0), (OrthonormalityConditionWeight 1.0), (PropernessConditionWeight 2.0). The number of iterations for each resolution is set to 500 to ensure sufficient number of iterations for convergence. At each iteration, 2000 samples are randomly selected to compute the MI of the fixed and the moving image. A stochastic gradient descent optimizer is used to find the optimal parameter $\hat{\mu }$ of the B-spline transformation field, which defines the best transformation of the moving image (the diagnostic image) to the fixed image (the intra-operative images). The registration is performed on an AMD Opteron core, 2.8GHz, 64 GB RAM on a Linux cluster.

### Evaluation metrics

Three quantitative metrics were used to evaluate the accuracy of registration method. The first metric is the Dice coefficient which measures the overlap between a segmentation of the liver in the diagnostic image and that in the intra-operative image after the registration. The segmentation of the liver in the diagnostic image is transformed to the intra-operative image using the transformation result of the registration. The Dice similarity coefficient (DSC) is computed as follows:

$$\text{DSC}\left(X,Y\right)=2\frac{\left|X⋂Y\right|}{\left|X|+|Y\right|}\;,$$ (4)

where *X* and *Y* present the two segmentations, and |.| denotes the number of voxels inside the segmentation. Secondly, we use the mean surface distance in 3D (MSD) to evaluate the distance between the surfaces of the liver segmentations after registration. The MSD is defined as follows:

$$\text{MSD}\left(X,Y\right)=\frac{1}{\left(n_{X}+n_{Y}\right)}\left(\sum_{i=1}^{n_{X}}d_{i}+\sum_{j=1}^{n_{Y}}d_{j}\right)\;,$$ (5)

where *n*_*X*_ and *n*_*Y*_ represent the number of voxels on the two segmentation surfaces correspondingly, and *d*_*i*_, *d*_*j*_ are the closest distances from each voxel on the surface to the other surface. In practice, the surface is determined by eroding with a kernel of one voxel.

**Fig 5:**
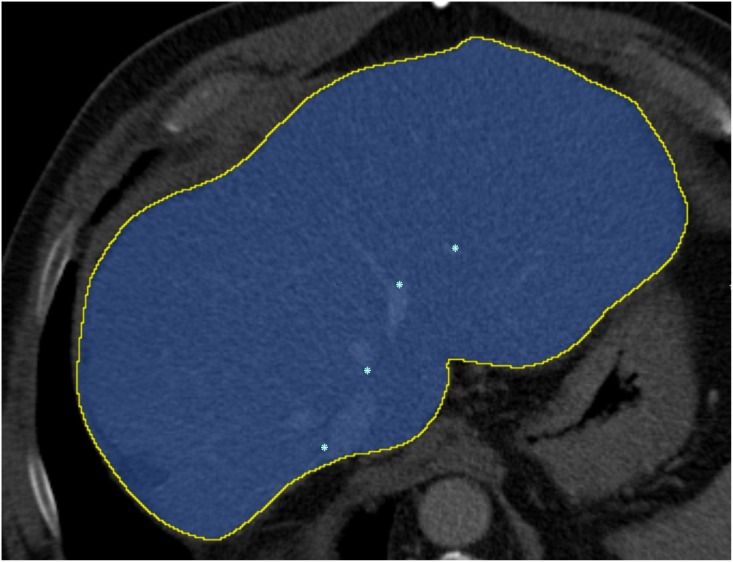
The manual liver segmentation and the landmarks inside the liver.

In order to measure the accuracy of the registration inside the liver, corresponding landmarks are used as the third evaluation metric (see Fig 5). 10-15 pairs of anatomical landmarks at the corresponding liver vessels (in case the intra-operative image has contrast agent) and surgical clips are annotated by two experts (radiologists). After registration, the mean corresponding distance (MCD) between the transformed points and the corresponding points is calculated as follows:

$$\text{MCD}\left(A,B\right)=\frac{1}{n}(\sum_{i=1}^{n}|a_{i}-T\left(b_{i}\right)\left|\right)\;,$$ (6)

where *n* is number of pairs of landmarks; *a*_*i*_ and *b*_*i*_ denote landmarks in diagnostic image *A* and intra-operative image *B* correspondingly; and *T* is the transformation from image *B* to image *A*.

The first stage (non-rigid) registration is the most challenging in our approach; all three metrics are used to evaluate this registration. Additionally, the DSC and MSD metrics are used for the limited field-of-view intra-operative images; MCD was not possible for these images, because of a lack of suitable landmarks.

### Registration results

**Fig 6:**
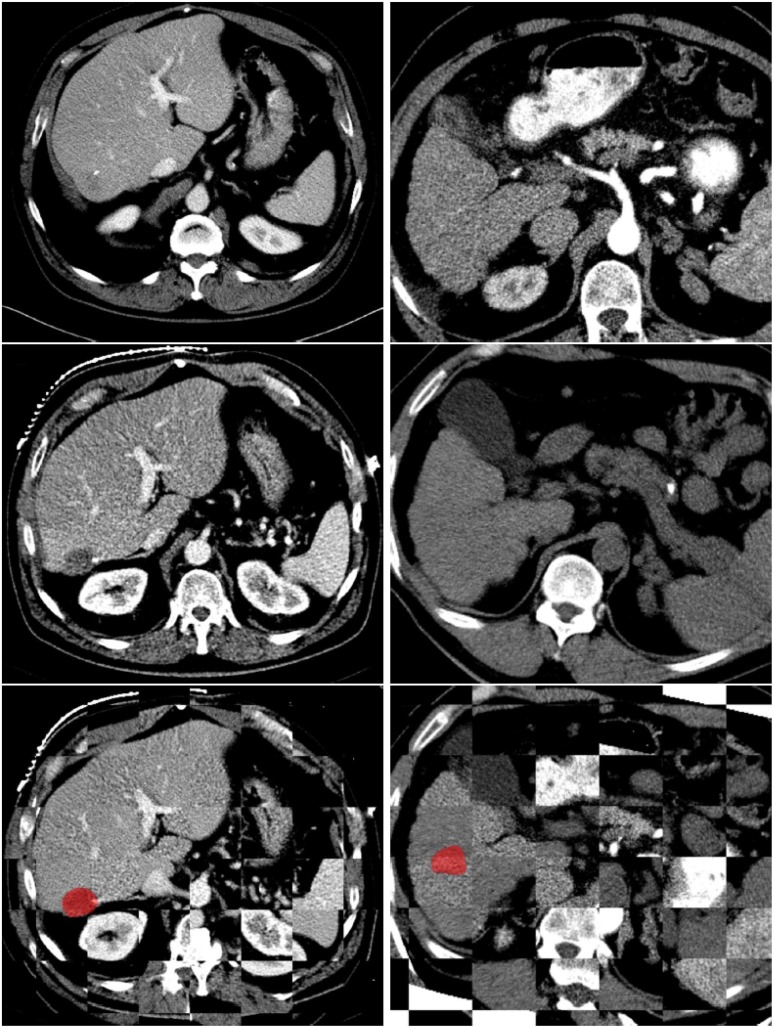
Two examples of successful registrations between diagnostic image and intra-operative image with the tumor (in red): The top row shows the diagnostic images; the second row shows the intra-operative images; the third row shows the fused images. The left column is an example of a contrast enhanced intra-operative image and the right column is an example of a non-contrast enhanced intra-operative image with 30 degree-rotation. The registration method only computes the metric within the liver, thus the images do not match outside the liver.

**Fig 7:**
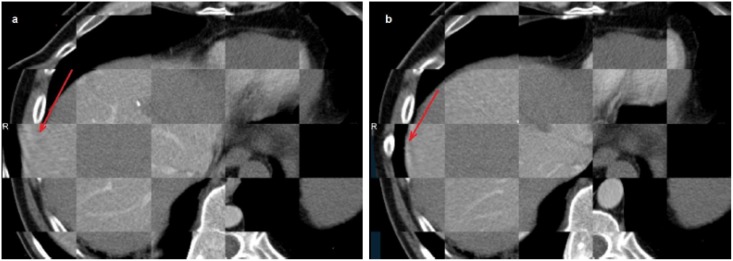
The effect of the rigidity term (from Eq 1) a) Registration without rigidity constrain, yielding large incorrect deformation (red arrow). b) Registration with rigidity constrain in the refined stage.

**Fig 8:**
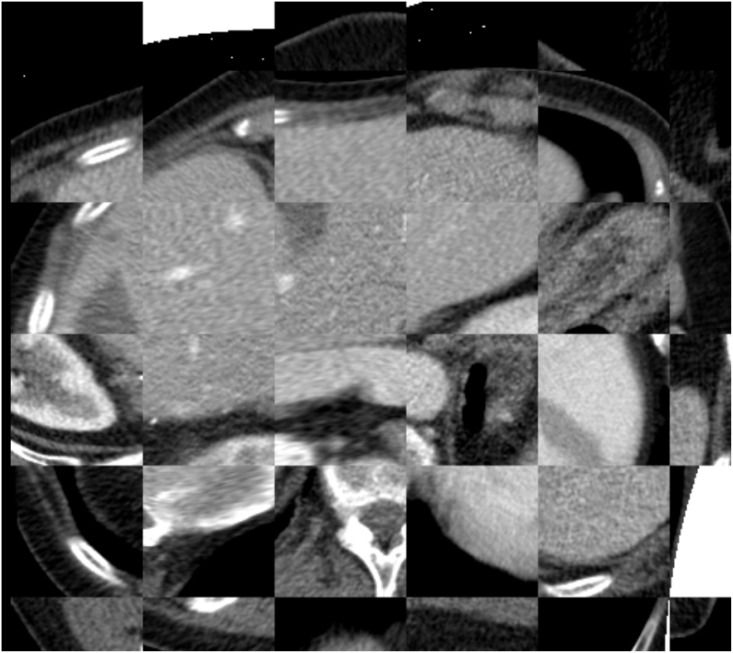
Example of an unsuccessful registration.

In a first experiment, we evaluate the registration performance of the first stage, i.e. the diagnostic CT image to the initial intra-operative image (the method described in section II) using the parameters described in section III.C. A pilot experiment was performed on the 5 random data sets with visual evaluation; and subsequently, we ran the software with fixed parameter settings on the 19 remaining data sets. In 13 of 19 cases the first stage registration was sufficiently accurate (determined by visual inspection with the DSC > 80% and MCD < 10 mm). The mean accuracy of registration of the successfully registered 13 cases is summarized in Table 1. The refinement step was applied on the remaining 6 cases. A user annotated 2—3 points to define the mask C (see Fig 4), which took around 1-2 minutes on average. Using this user input, an additional three cases were successfully registered. The evaluation result of these 3 cases using the metrics in section Evaluation metric (DSC, MSD, and MCD) is showed in Table 1. The remaining three cases remain with the DSC are lower than 80% are unsuccessfully registered. All of the three cases have large rotation (> 50 degree). Two examples of successful registrations in the first step are shown in Fig 6; an example of successful registration in the second step is show in Fig 7; and an example of unsuccessful registration is shown in Fig 8.

**Table 1:** Registration evaluation 16 cases in the first stage.

	13 cases	3 cases		All 16 cases
Metric	Non-rigid	Non-rigid only	Refined Non-rigid	Non-rigid
DSC (%)	91.4 ± 2.8	71.6 ± 14	90.3 ± 2.1	90.8 ± 2.9
MSD (mm)	4.4 ± 1.5	11.3 ± 4.2	5.1 ± 2.4	4.6 ± 1.8
MCD (mm)	4.7 ± 1.9	10.2 ± 4.6	6.4 ± 3.8	5.3 ± 2.5
Running time (min)	10-15	10-13	20-25	10-25

**Fig 9:**
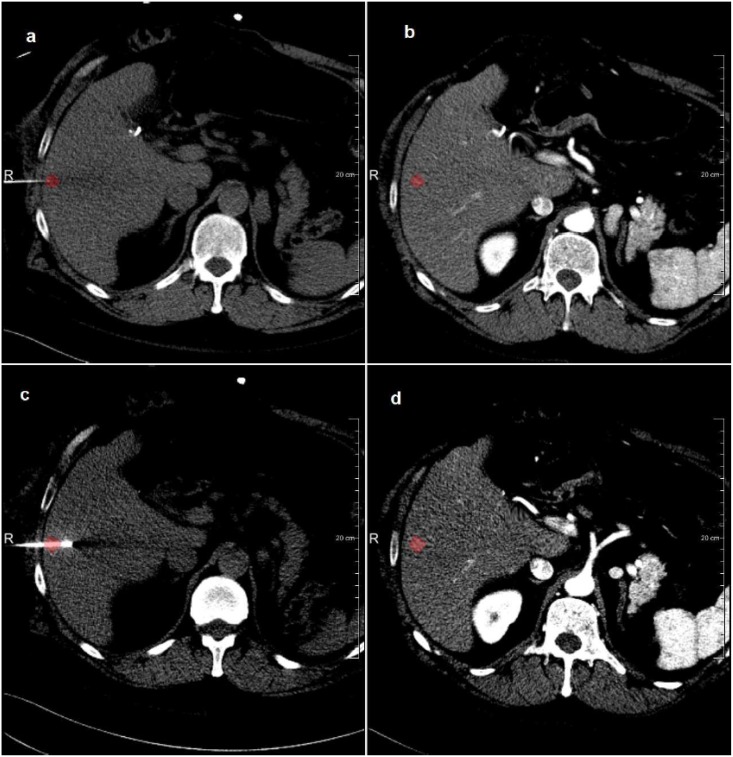
Example of tumor visualization in the intra-operative image after registration to the diagnostic image with the tumor (in red): a) the initial intra-operative image; b) the transformed diagnostic image to the initial intra-operative image; c) the next fame of intra-operative image; d) the transformed diagnostic image to the current intra-operative image.

A t-test between the registration accuracies in the two groups, non-contrast enhanced group (9 cases) and contrast enhanced group (7 cases), using DSC and MSD gave p-values 0.16 and 0.22 correspondingly, showing that there are no statistically significant differences between the two groups. A t-test on the registration accuracy between the rotation group (6 cases) and non-rotation group (10 cases) 0.002 For DSC, 0.0018 for MSD, demonstrating that rotation has significant influence on the registration accuracy. Next, we ran the second stage, i.e. the rigid registration between image F and image N followed by a non-rigid registration between image D and image N, which as initialized with the combined results of the registrations of image D to image F and image F to image N. This was only performed on the 16 datasets that successfully registered in the first stage. The results of this second stage registration are in Table 2, as well as the results if the second stage was left out, ie. if image F and image N were concatenated, without a subsequent registration and. An example of registration of the diagnostic image to a sequence of intra-operative images is shown in Fig 9.

**Table 2:** Registration evaluation of 16 cases in the second stage.

Metric	Concatenated only	Non-rigid
DSC (%)	83.9 ± 7.5	90.1 ± 3.6
MSD (mm)	6.8 ± 4.2	5.2 ± 2.6
Running time (min)	7-15	12-21

### Intra-observer variation

We also investigated the intra-observer variation in manually annotating the landmarks. This was done on the 11 datasets with contrast agent. To this end, the observers were asked to repeat their annotation in the interventional image, after presenting them the annotated landmark in the diagnostic image. The average distance between the original and repeated landmark annotations were 1.9 mm and 2.1 mm for both observers respectively.

## Discussion

In this study, we investigated a non-rigid registration framework to align diagnostic CT images to intra-operative CT images for CT-guided RFA of liver tumors. To be able to deal with intra-operative images with only a few slices, the registration was performed in two stages: a first stage in which the full diagnostic image was non-rigidly registered to the first intra-operative CT image, and a second stage where this registration, and a rigid registration between the first and current intra-operative image, were used to initialize a non-rigid registration between the diagnostic and current intra-operative image. The method also allows for manual interaction in case the non-rigid registration in the first stage did not give a good alignment. The approach was evaluated on 19 clinical datasets using three different metrics: DCS, MCD and MCD and compared with rigid registration method (see Tables 1 and 2). The results show that the proposed method achieved better registration accuracy than rigid registration. 13 of 19 cases were successfully registered (68%) without any correction, and another 3 were successfully registered using the user-defined local rigid areas, which improved the rate of successful registration to 84%. After correction, DSC was 90%, and MSD 5.1 mm and MCD were 6.5 mm. The 3 remaining cases, where the registration method failed to accurately register the images, are the cases with large liver deformation, caused by large rotation. The DSC of these three cases are 48%, 76% and 79%. The following intra-operative image registration resulted in a DSC of 90% and MSC of 5.2 mm, which shows that the largest registration errors are made in stage 1, and that no large errors are introduced in the second stage. When only the rigid registration from stage 2 is used, the DSC is 84% and MSD is 6.8 mm, which indicates that the non-rigid registration is required. The t-tests indicate that rotation affects the registration accuracy significantly and the contrast agent does not have a significant impact on the registration result. In other words, contrast agent might be omitted with this technique. Note that the clinical motivation for rotating the patient is to have sufficient access for e.g. ultrasound imaging, when using CT only, rotation is much less frequently required. For prospective evaluation of the proposed method, the number of cases with rotation can be decreased, and thus the success rate may be increased.

Interpretation of the error metrics should be done with care. First, the relatively large slice thickness of the CT datasets will lead to discretization effects in the error of the landmark annotation. Additionally, the intra-observer variation of approximately 1.9 mm and 2.1 mm demonstrates the difficulty in pinpointing landmarks for the evaluation, and is also a quantification of the in-plane error of the annotations. Both the in-between slice discretization error and the intra-observer error will be part of the registration error quantification, which is also confirmed by the visual inspection, which shows well aligned livers. Therefore, also taking into account the commonly used ablation margin of 10 mm in clinical practice, we are convinced that the registration-based integration of the diagnostic information in the intervention is a valuable add-on to the current practice of CT-guided RFA. Compared to other studies on registration of liver images our study has comparable results. Elhawary et al (2010) registered diagnostic contrast-enhanced MRI to intra-procedural CT of the liver in cryoablation. A TRE accuracy of 4.1 mm and a DSC of 97% were reported. Our TRE of corresponding landmarks is 5.3 ± 2.5 mm, and our DSC is 91 ± 2.9%. Note that our intra-operative images were acquired with lower tube current and tube voltage. Comparing these results to our previous study using the same framework with the pre-operative RFA image and the post intra-operative RFA image, which has a DSC of 92.2%, a mean distance between the liver segmentation boundaries of 2.51 mm and a MCD of 2.91 mm, we conclude that registration to the intra-operative images is more difficult. The less protocolized imaging (no breath-hold), lack of contrast agent and patient rotation may be the cause of these differences.

Our study has some limitations. First, we only use data from a single center with limited number of CT scanners. We are, however, confident that similar results will be obtained using imaging data from other CT system vendors, as image acquisitions are protocolized, and Hounsfield units present physical properties. Second, because of limited size of the intra-operative images with the needle in place, we were not able to annotate landmarks to be used for the evaluation. However, based on the visual alignment of the liver boundary (see Fig 9 for an example), we expect that the accuracy of the registration is similar to the accuracy of registration between diagnostic image and the initial intra-operative image.

Finally, the registration running time is from 7 to 20 minutes which is insufficiently fast for direct use in clinical practice. This problem can be solved by using multi-threaded programs and/or using GPU. In the future, we intend to investigate more semi-automatic approaches that may be used to address the unsuccessful registration, and also we intend to evaluate the registration framework in a prospective interventional setting.

## Conclusion

In conclusion, we have developed and evaluated a two stage registration approach to align diagnostic CT images to intra-operative CT images for CT guided liver cancer RFA treatment. The method had a success rate of 84%, a registration accuracy of 91% in terms of DSC, 4.6 mm in MSD and 5.3 mm in MCD. These results show that the registration approach is a promising tool for improving RFA needle positioning in minimally invasive treatment of liver cancer.
